# What emotions do male prisoners experience in the lead-up to suicide and violence? A participatory visual method study

**DOI:** 10.1080/14789949.2023.2199717

**Published:** 2023-04-28

**Authors:** Laura Hemming, Peer Bhatti, Gillian Haddock, Jennifer Shaw, Daniel Pratt

**Affiliations:** a Division of Psychology and Mental Health, School of Health Sciences, Manchester Academic Health Sciences Centre, University of Manchester, Manchester, UK; b Greater Manchester Mental Health NHS Foundation Trust, Manchester Academic Health Sciences Centre, Manchester, UK

**Keywords:** Suicide, violence, emotion regulation, participatory visual methods

## Abstract

Rates of suicide and violence are higher amongst male prisoners than the general population. This study aimed to explore the emotional experiences of male prisoners in the distal and immediate lead-up to acts of suicide and violence. Nine male prisoners created drawings of their emotions in the lead-up to an act of suicide and/or violence. Accompanying verbal interview data was collected to explore the narrative of these drawings. Polytextual thematic analysis was conducted on both the visual and audio data. Three themes were found. ‘The outside picture’ depicted the emotions that male prisoners exhibited externally. ‘The inside picture’ illustrated the internal emotions felt by male prisoners which were often complex and abstract. ‘The complexity of the picture’ denotes the complicated relationship between emotions and suicide/violence. Male prisoners experience a range of emotions in the lead up to acts of suicide and violence, with a similar set of emotions being experienced immediately prior to both suicide and violence. This study has illustrated the benefits of using a novel and creative methodology, and has demonstrated that future research with male prisoners could benefit from adopting a participatory visual methodology.

## 1. Introduction

This study adopts the following definition for suicide ‘Suicide is death caused by injuring oneself with the intent to die.’ (CDC, n.d.). This study adopts the National Institute of health and Care Excellence (NICE) definition for violence: ‘Violence and aggression refer to a range of behaviours or actions that can result in harm, hurt or injury to another person, regardless of whether the violence or aggression is physically or verbally expressed, physical harm is sustained, or the intention is clear.’ (NICE, 2015).

Using the definitions above, UK wide data show that suicide and violence are common in male prisons. In the UK, there were 86 self-inflicted deaths in 2021, representing an increase of 28% from the previous 12 months (Ministry of Justice, 2022). This takes the rate of self-inflicted deaths to 1.1 per 1,000 prisoners (Ministry of Justice, 2022). In the same year, there were 52,736 self-harm incidents, enacted by 11,236 individuals (Ministry of Justice, 2022). There were also 12,579 prisoner-on-prisoner assaults at a rate of 161 assaults per 1,000 prisoners, with 2,042 of these being classed as serious assault incidents (Ministry of Justice, 2022).

Outside of a prison population specifically, several theories of suicide centre on emotions as a key component in the lead up to both suicidal ideation and suicidal behaviours. For instance, the interpersonal-psychological theory of suicide posits that feelings of perceived burdensomeness and thwarted belongingness can lead an individual to consider suicide (Ribeiro & Joiner, 2009). The integrated-motivational-volitional theory of suicide states that feelings of defeat and humiliation (amongst other factors) can lead an individual to develop suicidal thinking (O’connor & Kirtley, 2018). It has also been proposed that emotions play a role in the development of suicidal ideation, though the transition from ideation to action depends on other factors (e.g. acquired capability for suicide, access to means) (Law et al., 2015).

Similarly, the most widely accepted model of violence, the general aggression model, proposes that in order for an individual to commit an act of violence, this is dependent on (amongst other factors) affective state (Anderson & Bushman, 2002). Emotions such as anger, rage and revenge have been frequently found to relate to violent behaviours (Allen & Anderson, 2017).

Given the strong theoretical and empirical link between emotions and both suicide and violence, it is imperative to explore how emotions are experienced by individuals in both the distal and immediate lead-up to acts of suicide and/or violence. Previous attempts have been made to explore such factors in the domain of self-harm in adolescents (Townsend et al., 2016), however no such attempt has been made to explore this within a male prison population, in which it is known that rates of suicide and violence are much higher than in the general population (Fazel et al., 2017). Understanding which emotions are experienced in the lead-up to suicide and violence, in a population known to be at a higher risk of these behaviours, is crucial for intervention and prevention efforts. Identifying the emotions experienced by prisoners will lead to clear treatment targets, both in those receiving individual therapy for suicide and/or violence, as well as more broad prevention efforts across the prison system (for instance, psychoeducation on how to regulate emotions identified as being related to suicide and/or violence).

Of even greater importance, is understanding the relationship between emotions and suicidal or violent behaviours amongst those who may struggle to identify and describe their emotions. Such an experience can be labelled alexithymia and is thought to comprise five main features (Taylor & Bagby, 2000); i) a difficulty in identifying one’s emotions ii) a difficulty in describing self-feelings verbally iii) a reduction or incapability to experience emotions iv) an externally orientated cognitive style and v) poor capacity for fantasising or symbolic thought. Indeed, itis known to affect around a third of those in incarceration (Louth et al., 1998; Parker et al., 2005; Zimmermann, 2006). Specifically, some male prisoners have been found to struggle to identify and describe their feelings (Hemming et al., 2021; Hemming, Bhatti, et al., 2020; Hemming, Pratt, et al., 2020). As such, traditional qualitative interview techniques may be of limited use in this population. Indeed, it has previously been found that male participants in particular may struggle to be forthcoming with emotional experiences which can lead to a lack of satisfactory data for researchers (Duncombe & Marsden, 1993; Presser, 2006).

Visual methodologies are therefore deemed particularly useful for accessing the experiences of those who have difficulties verbally articulating experiences. Art therapy, for example has been described as a modality that helps individuals to verbalise their thoughts and feelings, beliefs, problems and world views (Malchiodi, 2011). A previous randomised controlled trial found that 15 weekly sessions of art therapy led to a significant decrease in depression and a significant increase in locus of control in male and female prisoners (Gussak, 2009) highlighting its potential value in the prison setting.

The use of visual methodologies could bring a range of benefits to qualitative research. First, it is noted that images are often used as a stimulus to discover richer accounts of phenomena being studied (Lyon, 2020; Meo, 2010; Samuels, 2004; Wang & Redwood-Jones, 2001). Indeed, it has been noted that the parts of the brain that process visual information are evolutionarily older than the parts which process verbal information, and so images often evoke deeper elements of human consciousness than words (Harper, 2002). Second, visual methodologies can help to build rapport between researcher and participant as well as ensuring that interviews remain engaging whilst structured (Collier, 1957; Pain, 2012). Third, visual methodologies can help facilitate triangulation, whilst recognising that no single empirical method is capable of fully capturing a phenomenon (Patton, 2002).

Specific to this study, much previous research into alexithymia amongst prisoners has been quantitative in nature (e.g. Louth et al., 1998; Parker et al., 2005; Zimmermann, 2006). Such an approach to studying this phenomenon therefore omits detailed individual accounts of the experience of alexithymia. It is therefore crucial that qualitative research be conducted into the experiences of suicidal and violent prisoners in terms of their emotions. Visual methodologies represent an efficient way of accessing accounts of such experiences. This is due to the ability to use illustrations as metaphors to convey complex and intense emotional experiences that may otherwise be difficult to express due to limitations of emotional vocabulary (Affleck et al., 2013). It has been noted that there is always a degree of power imbalance present in qualitative interviews (Hill, 2013), and this may be amplified in the case of particular populations such as prisoners. It is argued that such a power imbalance may mean that traditional semi-structured interviews reflect a participant’s tendency to agree with researchers’ predisposed ideas about a topic (Duckett & Fryer, 1998). Visual methodologies, particularly those that are participatory (that is driven by the participant who produce images themselves for the research project either via photography or drawing), may go some way to redressing the power imbalance between researcher and participants. This is due to them encouraging the participant to take control of their participation by leading the direction and content of interviews (Bates et al., 2017; Oliffe & Bottorff, 2007). As well as empowering the participant in the research process, this might also enable new understandings of phenomena derived from the experiences and knowledge of participants, rather than researchers (Bates et al., 2017; Holloway & Galvin, 2016; Hurworth, 2004).

This study therefore aimed to use participatory visual methodologies to explore the emotional experiences of male prisoners leading up to acts of suicide and/or violence.

## 2. Methods

Detailed methodology for this study has been published elsewhere (Hemming, Bhatti, et al., 2020), however details pertinent to the current study are included below.

### 2.1. Participant selection

Participants were eligible to take part in this study if they were aged 18 or over and had been residing in a male prison in the North West of England for at least one week. Furthermore, eligible participants must had to answer yes to at least one of the following questions: *(1) Over the past three months have you thought about killing yourself? (2) Over the past three months have you tried to kill yourself? (3) Over the past three months have you thought about hurting somebody else? (4) Over the past three months have you tried to hurt somebody else?* Finally, eligible participants must be deemed to experience alexithymia, measured by having scored 52 or above on the Toronto-Alexithymia Scale (TAS-20: Bagby et al., 1994). Participants were excluded from the present study if they; did not possess sufficient mental capacity to give informed consent, were due for transfer or release within the next week or were assessed by the prison’s security department as too high risk due to security intelligence.

Participants either self-referred into the study and were then assessed for eligibility or were referred from a separate cross-sectional study (Hemming et al., 2021) which deemed the individual eligible for the current study. Participants who self-referred into the study did so through responding to adverts placed around the prison, which asked them to speak with a member of the safer custody team in each prison, who would then pass potential participants on to the first author. All potential participants were approached face-to-face by the first author through a meeting on the wing in which they resided. In this meeting, a detailed overview was given of the study, and for those who self-referred an eligibility assessment was undertaken. Purposive maximum variation sampling was used to obtain a varied set of participants who differed based on their alexithymia scores, whether they had experienced custodial suicide or violence or both, age and ethnicity. This was achieved by creating a recruitment matrix throughout the study, highlighting where further participants were needed to satisfy particular experiences.

Twenty individuals were approached to take part in the study. Of these five individuals declined the invitation due to: fears that the audio recording would be shared with others, concerns about participation affecting their parole and not feeling emotionally stable enough to participate at that moment in time. Fifteen individuals were recruited to take part in this study. Due to iterative changes to the topic guide, only eleven of these participants were offered the opportunity to draw their emotions. Early interviews included a researcher-driven methodology, whereby images of common emotions were given to participants to discuss. This approach was felt to be too far removed from the participants personal experiences of emotions, and so the visual methodology was revised to include participant drawings of emotions. Of the 11 participants offered the opportunity to draw their emotions, only nine agreed to take part in the drawing element of the interview. Informal reasons given for not wishing to draw emotions were a lack of confidence in drawing ability, and a fear of embarrassment. The total sample for the present study was nine.

### 2.2. Setting

All data were collected in two prisons in the North West of England; one was a category A local prison, and one a category C. Data were collected between August and November 2019. Interviews took place in a private location within the prison, with only the researcher (LH) and participant present.

### 2.3. Data collection

A participatory visual method design was employed for data collection (De Lange & Stuart, 2007). An interview guide was developed in collaboration with, and pilot tested by, a lived experience advisory group. Individuals were recruited to the advisory group on the basis of having lived experience of custodial suicide and/or violence, but did not need to have experienced alexithymia. The advisory group were separate to the individuals participating in the study, and provided input throughout several stages of the study including the design, analysis and reporting.

The topic guide contained questions, prompts and guides for the researcher to use. The interview began with the researcher asking the participant what they understood by the word ‘feelings’. Where it was considered the participant did not have a good understanding of what feelings were, a standardised definition was given to participants. Then,, the following questions were asked (amongst others):

1. Have you experienced any strong emotions lately? Can you tell me about a recent time? *(Probes: upset, happy, worried, disgusted, shame, angry, love)*
2. What is it like to feel like this? *(Probe: physical reactions, thoughts)*
3. If you could draw these emotions, what would they look like? Would you like to have a go at drawing these for me? *(Probes for meaning of drawings)*
4. Can you tell me about a recent time that you felt suicidal and/or violent?
5. What emotions did you feel in the lead up to this? *(Probe: minutes/seconds before, hours before, days before)*
6. If you could draw these emotions, what would they look like? Would you like to have a go at drawing these for me? *(Probes for meaning of drawings)*

Participants were provided with a range of materials with which to complete their drawing – including coloured pens, pencils and paper. Interviews were audio recorded and transcribed verbatim and lasted 65 minutes on average. Drawings produced as part of the interview were retained as research data. Extensive field notes were taken during and after interviews, which focussed both on the content of the interview, as well as environmental and interpersonal factors that may have impacted the data collection.

### 2.4. Data analysis

A polytextual thematic analysis was conducted following the steps outlined by Gleeson (2012). The steps taken are broadly similar to those outlined in Braun and Clarke’s thematic analysis (Braun & Clarke, 2006). First, researchers independently viewed the images repeatedly singly, in groups, serially and in as many different orders as possible, whilst making notes in a reflective log as well as noting any potential themes. This stage was focussed on identifying similarities and differences between the images, and sorting the images by their common components. Where a potential theme appeared to occur more than once, all material relevant to that theme were collected and a brief description or definition of the proto-theme was created. All other images were then searched for evidence of the newly developed proto-theme, and the description was revised if necessary. This process continued until no further distinctive themes relevant to the research question were identified. Finally, higher-order themes which connect themes together were searched for.

Accompanying verbal data were used to supplement the meanings behind the drawings, and thus these were incorporated into the polytextual thematic analysis described above. Verbal interview data were organised using NVivo version 11 software.

Two individuals were responsible for analysing the data (first and second authors). This ensured that a mixture of perspectives were included at the data analysis stage, including the perspectives of those with lived experience of custodial suicidality and violence.

### 2.5. Reflexive statement

A reflexive statement is advised to make explicit the background of those analysing the data, in the hope of making transparent any biases which may have guided interpretations of both visual and audio data.

LH is a 31-year old, White British female with a PhD in Psychology and Mental Health. She has worked in mental health research for over eight years and has a wealth of previous experience of leading and contributing to thematic analyses on a range of mental health related research projects.

PB is a 69-year-old, White British male with a BA (Hons) degree in modern middle eastern history. He has previously served a five-year sentence in prison, during which time he became a ‘Listener’ – a prisoner trained by the Samaritans to help other prisoners at risk of suicide and self-harm.

GH is a White British female with over 30 years experience in the field of mental health. She has a PhD and professional qualification in clinical psychology and is Professor of Clinical Psychology at the University of Manchester (UoM). She also holds an honorary appointment at Greater Manchester Mental Health (GMMH) NHS Foundation Trust. She is a co-Director of the Suicide and Safety Research Unit, a collaboration between the UoM and GMMH. GH’s professional expertise lies primarily in psychosis, suicide, forensic psychology and substance misuse.

JS is a White British female with a PhD in Psychology and Mental Health. She is a Professor of Forensic Psychiatry, a Consultant Forensic Psychiatrist and academic lead for the Offender Health Research Network. She is also a panel member for the independent Advisory Panel for deaths in custody.

DP is a 49-year-old white, British male with a PhD as well as a Clinical Psychology professional Doctorate. He is a clinical senior lecturer at the University of Manchester and holds an honorary appointment as a Clinical Psychologist at Greater Manchester Mental Health NHS Foundation Trust. DP’s professional experience lies primarily in development and evaluation of interventions for suicide prevention, with a particular interest in forensic populations.

## 3. Results

### 3.1. Participant characteristics

All participants identified as male, and the average age was 34 years old (range 27 to 43 years). The majority of participants identified as White British (*N* = 8). The average alexithymia score was 75.7 (SD = 9.6), which sits above the suggested cut-off score of 61 indicating ‘high alexithymia’ on the TAS-20. A total of seven participants had experienced thoughts of suicide in the three months prior to participating, two had experienced suicide attempts, eight had experienced thoughts of violence, and two had attempted violence against another. The majority of participants (*n* = 6) experienced either thoughts or behaviour related to *both* suicide and violence.

### 3.2. Thematic analysis

Participants produced between one and four images each, leading to a total of nineteen images. Three higher-order themes were identified; ‘the outside picture’, ‘the inside picture’ and ‘the complexity of the picture’. ‘the outside picture’ contained four subthemes; anger, sadness, anxiety and positive emotions. ‘the inside picture’ contained two subthemes; inner turmoil and an absence of emotions. ‘the complexity of the picture’ depicts how these emotions relate to one another and also to the experiences of suicide and violence.

### 3.3. The outside picture

This theme referred to drawings which depicted outward displays of emotions. Participants often chose to draw physical, behavioural responses to emotions or behaviours that one might enact due to experiencing particular emotions. Participants also often drew situations in which they pictured themselves experiencing this emotion. All drawings depicted tangible, concrete examples of manifestations of emotions, and emotions ranged from negative (anger, sadness and fear) to positive (happiness, hope and love).

#### 3.3.1. Anger

**Figure 1.**
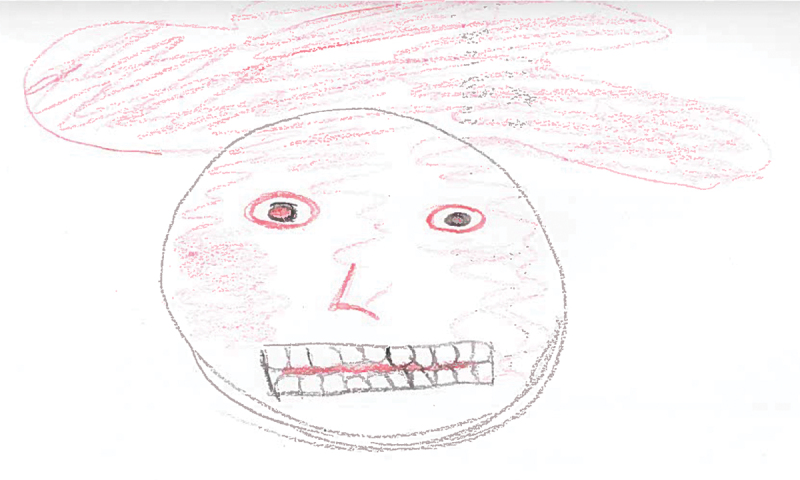
WQ10 drawing of anger and hatred.

Six participants created drawings which depicted anger, or related emotions such as frustration or hatred. These six drawings possessed a number of common features. For instance, participants often used the colour red to portray anger. A number of participants illustrated the physical responses to this emotion such as images of people frowning, clenching their teeth or their eyes turning red. A clear commonality was the drawing of faces, which is perhaps unsurprising given their centrality in our display of emotions. Three participants also attempted to visually demonstrate the ‘mist’ that they experience which was described as making things foggy, and therefore makes it difficult to see what your next move is (see Figure 1 for an example). Finally, several participants purposefully drew only themselves in the picture – one participant stated this was because anger can make you feel lonely and make it difficult to form relationships with others.

“*It’s a cloudy sort of view of what’s going on type thing. You don’t know what you’re walking into, you don’t know what’s coming because you can only see sort of just in front of you … It’s sort of like a whirlwind grey mist.” (WQ10)*

*“It’s just lonely as well. You can’t let anybody in because you might be angry with them, you might upset them, they might make you worse*, *you know it’s difficult.” (WQ06)*

#### 3.3.2. Sadness

**Figure 2.**
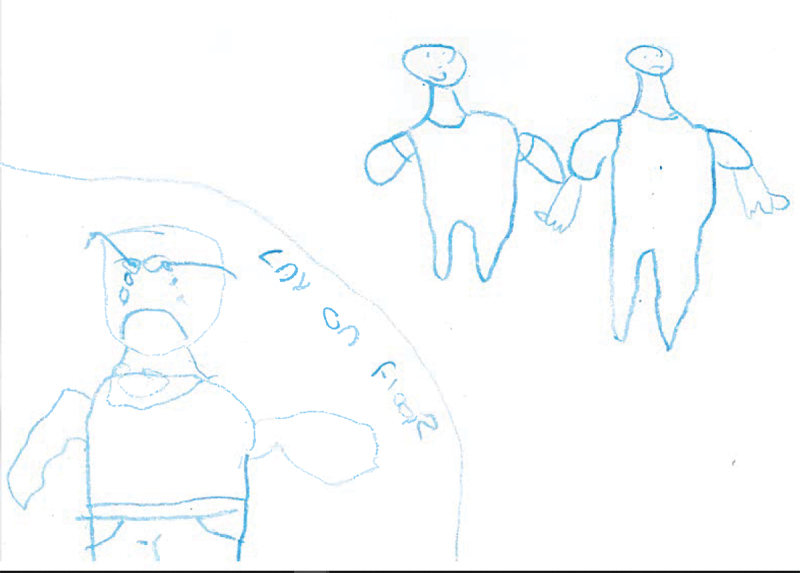
WQ06 drawing of sadness.

Six participants created drawings which depicted outward displays of sadness, either on its own or in tandem with other emotions. Again, participants visually depicted physical responses to sadness such as crying, an ‘upside down smile’ and feeling tired. Participants also attempted to visually convey the feeling of being emotionally drained, and feeling like they can’t be bothered. Participants made deliberate reference to their positioning to other individuals, for instance with the picture below (Figure 2) demarcating the participants from others in the prison who are placed closer to each other. One participant visualised sadness as a broken heart.

#### 3.3.3. Anxiety

**Figure 3.**
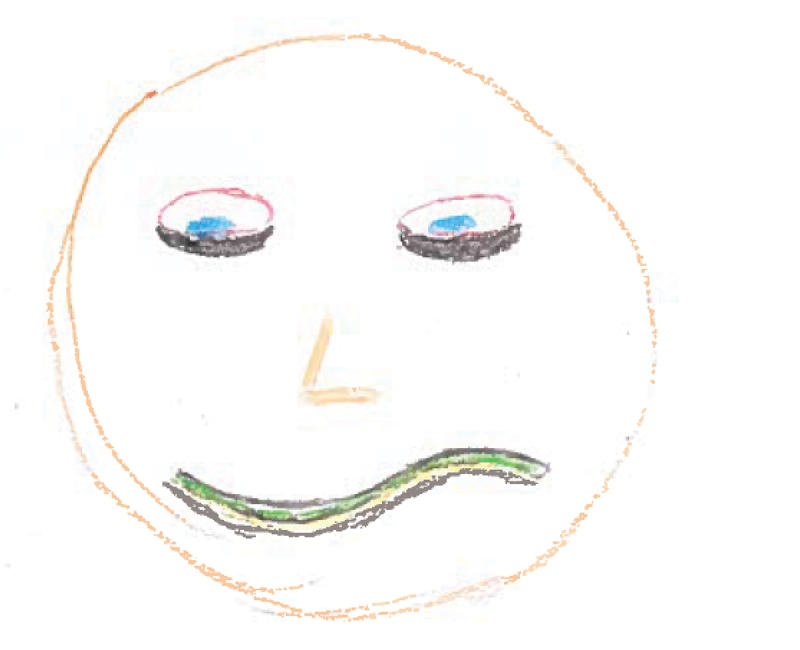
WQ10 drawing of worry.

Five participants created drawings of anxiety or related emotions such as fear, worry and nervousness. Participants focussed on physical depictions of this emotion such as bags under the person’s eyes due to not sleeping well and a ‘wobbly’ mouth (see Figure 3 for an example). This emotion was drawn (and discussed) more closely in relation to specific situations than any of the other emotions, with participants frequently discussing experiences such as being bullied whilst in prison. Again, participants deliberately included only themselves in the picture due to the acknowledgement that anxiety could make you feel lonely. One participant noted that externally, the individual would look ‘normal’ because they would not want to display anxiety which would suggest weakness in the prison environment.

*“Hopefully [my face looks] just normal. It’s like open, smiley, non-aggressive … I’ll try to hide the fact that I’m worried, because if you show fear or weakness people can kind of thrive on that.” (WQ06)*

#### 3.3.4. Positive emotions

**Figure 4.**
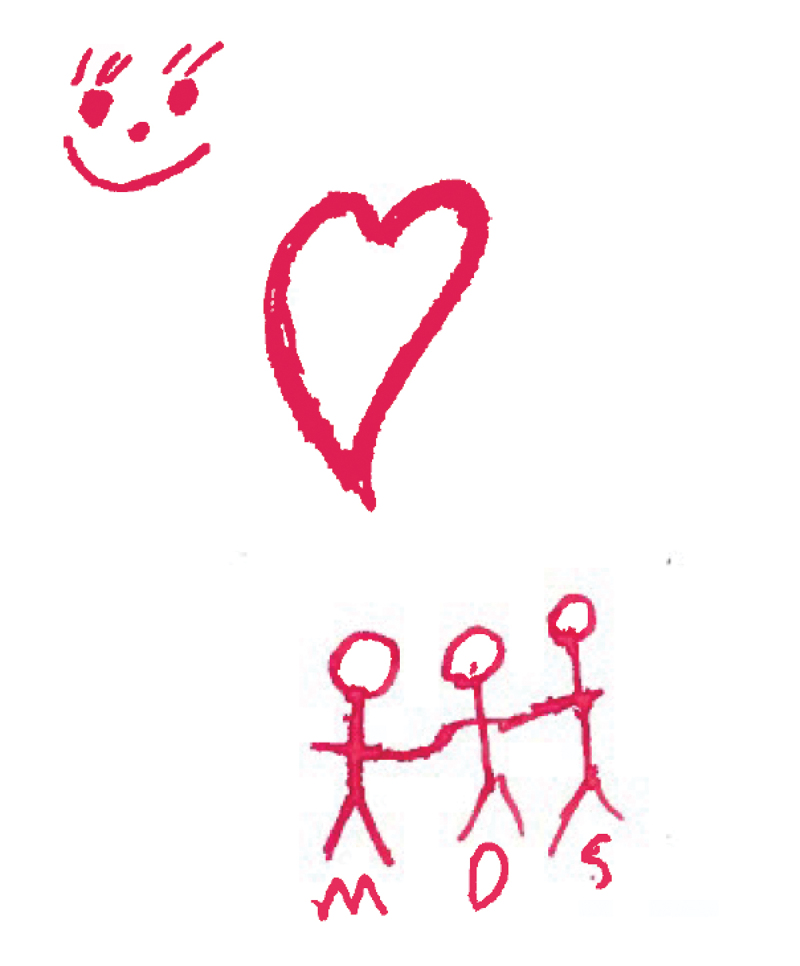
WQ07 drawing of love and happiness.

Somewhat surprisingly, five participants created drawings of positive emotions such as happiness, hope and love. Indeed, one participant felt that this emotion was the ‘easiest’ to draw out of all emotions. Participants made clear that positive emotions were very separate from negative emotions – whereas drawings of anger, sadness or anxiety frequently depicted more than one emotion, drawings of positive emotions were often solely devoted to that emotion. Participants used colour imagery to portray positive emotions such as using the colour red to indicate love and the colour green to represent a bright day and positive thinking. Participants also drew situations that they frequently related to happiness such as sunshine, going fishing and being with their children. See Figure 4 below for an example of a drawing of positive emotions.

### 3.4. The inside picture

There seemed to often exist a direct tension between the emotions that participants drew external examples of, and how this matched to what participants experienced internally. For instance, the clear delineation of emotions in the theme ‘the outside picture’ indicates a good understanding of emotions and how each of these feels. However, the theme ‘inside picture’ suggests that participants may have experienced significant difficulty identifying or distinguishing their emotions, particularly in the lead up to suicide or violence.

#### 3.4.1. Inner turmoil

**Figure 5.**
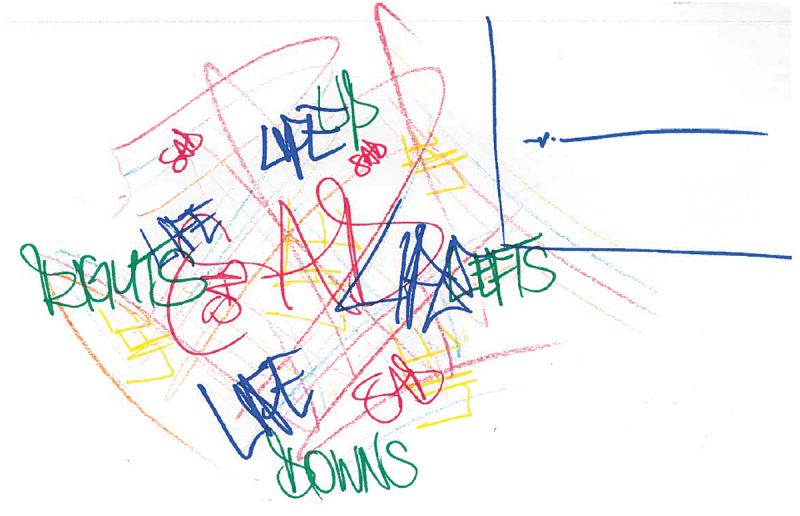
WQ11 drawing of confused emotions.

Four participants created pictures which have been categorised as displaying inner turmoil. These images were characterised by feeling several distressing emotions all at once, in combination with physical feelings. These were considered to feel overwhelming and all-consuming, which was indicated by the fact that these images often covered the entire page, leaving no blank space on the page. In Figure 5 below, the participant has used various colours and textures as well as a combination of words that felt salient to the individual, to depict this overwhelming ‘whirlpool’ of emotions. Participants stated that feeling like this was very confusing, and therefore created difficulties in trying to understand these emotions and communicate them to others.

#### 3.4.2. An absence of emotions

**Figure 6.**
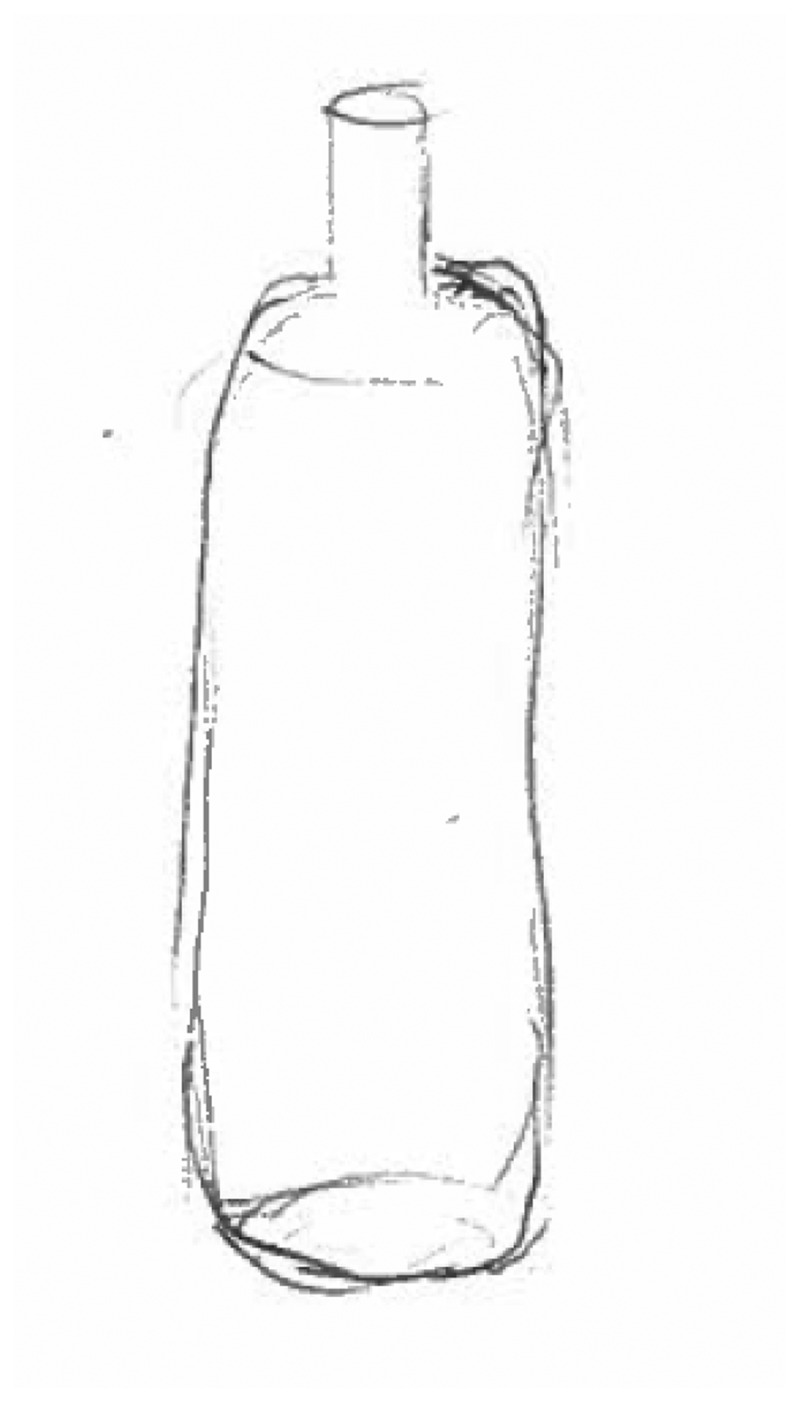
WQ12 drawing of absence of emotions.

In stark contrast to the inner turmoil described above, three participants described feeling an absence of emotions. Such an experience was characterised by feeling ‘empty’, ‘void’ or ‘flatlined’. Interestingly, one participant drew *both* the experience of inner turmoil as well as an absence of emotions. Some participants described this feeling as desirable – as it could provide relief from the turmoil described above. Participants depicted this feeling in different ways. One participant used a biro pen to cover the entire page in black ink, explaining that there is ‘nothing there’. Another (see Figure 6 below) drew an empty bottle, explaining that he felt there were ‘no dregs left’. Participants often stated that this was the emotion they felt immediately before either hurting themselves or hurting somebody else. This suggests that although for some this absence of emotions could be relieving, for others it could lead to distress, indicated by the preference to inflict physical pain on oneself or others than to be left feeling nothing.

*“It’s like I’m empty. The only thing I can think of is like an empty bottle or something. ‘Cause at the time I don’t recognise my feelings, ‘cause I start smashing things and I don’t realise what I’m doing until someone tells me. So, it’s like there’s nothing there, there’s nothing at the bottom of the bottle. The bottle’s empty, there’s no dregs in there.” (WQ12)*

### 3.5. The complexity of the picture

Out of the nineteen images produced, eleven (58%) of the drawings included more than one emotion, which reflects how common it is to experience more than one emotion at the same time. Whilst some combinations of emotions could be considered understandable (e.g. hatred and anger, anger and frustration), there were also interesting combinations which spanned both positive and negative emotions (e.g. happy and nervous).

Interestingly, the same emotions were drawn both in tandem with images of violence and images of suicide or self-harm. For instance, whilst anger was frequently drawn in relation to violence, damaging property or verbal aggression, it was also drawn in relation to self-harm. Similarly, whilst sadness was often drawn in relation to suicide, it was also drawn in relation to violence towards others.

**Figure 7.**
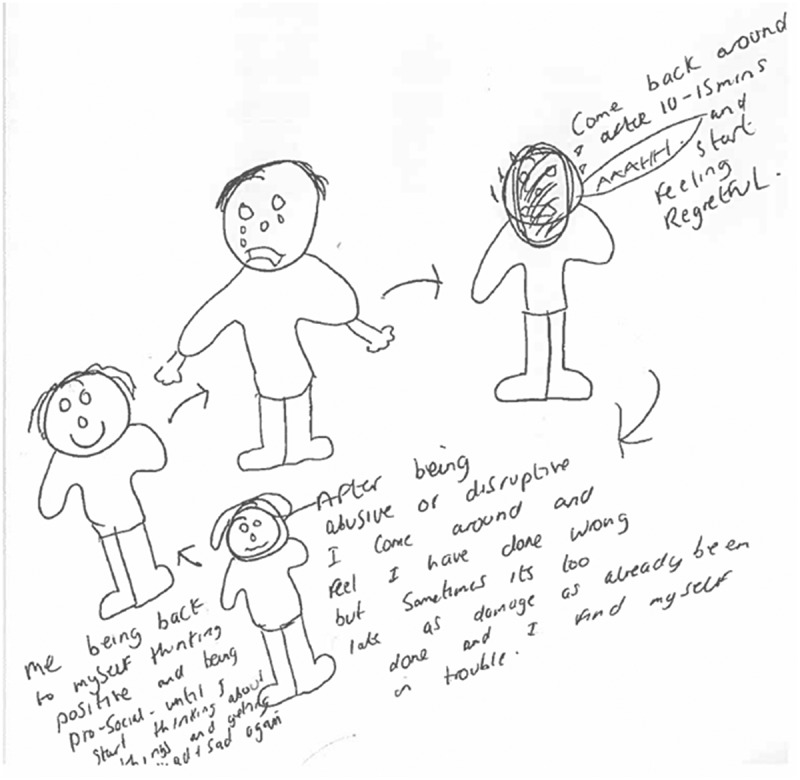
WQ13 drawing of a cycle of emotions.

Finally, two participants visually depicted the relationship between different emotions they experienced. For instance, one participant drew the emotions of anger, sadness, fear and happiness and then explained that happiness was very much separate to the other three emotions. He elaborated that fear was also at the root of the other two negative emotions – for instance, if he was sad, he would also feel fearful that somebody might see him feeling sad which would suggest a weakness. And if he was feeling angry, he would also be fearful of what he might to do somebody else. The other participant explained not just the relationship between emotions but also their chronology (see Figure 7). He explained how he could begin by feeling happy, but then if he began ruminating on situations this could lead him to feeling sad. This sadness then led to him ‘putting on a mask’ which was associated with feelings of anger, as well as behavioural displays of anger (‘ranting and raving’). This then leads to him feeling regretful for his actions. The participant explained that the cycle was adaptable; he could sometimes skip the sadness part and jump from happiness to anger very quickly. Alternatively, the cycle could be reversed; if he was feeling sad and he talks about it with somebody and this was deemed to ‘go well’, then this could lead to feelings of happiness. However, if the conversation was not deemed to ‘go well’ then this could lead to feelings of anger.

*“I feel frightened that somebody will see me being upset because then they’ll take the mick. And I’m obviously frightened with my anger because I don’t know why it’s just all of a sudden it’s becoming violent, man. And it’s not just little bits of violence, you know like a little scrap, it’s full out hurting people and full out taking people hostage.” (WQ15)*

*“Every time I get mad, this is like a cycle … Like if I’m feeling sad or just say I’ve been arguing with somebody on the phone like this mask will come on where it’s just me being negative and angry and I start ranting and telling people to F off and all this and that. And then I’ll go back to my cell and I’ll sit there and this is always the same, I feel, I start off feeling sorry for myself … I could probably be happy for a while. And then I just start thinking about things then I get sad again then I start turning into an angry person. It’s just a cycle.” (WQ13)*

## 4. Discussion

### 4.1. Summary of results

This study aimed to explore the emotions experienced by male prisoners in the lead-up to acts of suicide and/or violence. Using participatory visual methodologies, this study found that male prisoners demarcate between those emotions displayed externally and those emotions experienced internally in the lead-up to suicide and/or violence. This supports previous qualitative research which has found that male prisoners tend to ‘mask’ their emotions, in order to deal with the physical and psychological challenges posed by the prison environment (Crewe, 2012, 2014; De Viggiani, 2012; Jewkes, 2005; Karp, 2010). This also suggests that these results may be due to the state of prisons, rather than due to the state of the individuals, supporting a deprivation argument as opposed to an importation argument (Dye, 2010).

The internal emotions that prisoners experienced in the lead-up to suicide and/or violence were presented as complex, confusing and abstract. This supports a previous qualitative study conducted by the current authors which found that, due to the complexity of emotions experienced, male prisoners struggled to identify and communicate these emotions to others (Hemming, Bhatti, et al., 2020). Indeed, rates of alexithymia (defined as a difficulty with identifying and communicating emotions), are known to be higher in prisoners than in other populations (Louth et al., 1998; Parker et al., 2005; Strickland et al., 2017; Zimmermann, 2006).

Specifically, the internal emotions depicted in this study fell into one of two categories; ‘inner turmoil’ and ‘an absence of emotions’. Several previous theories suggest that each of these affect states is related to both suicide and violence (Bushman et al., 2001; Jakupcak, 2003; Kaplan et al., 1998; Maltsberger, 2004; Moskowitz, 2004; Porteus & Taintor, 2000; Shneidman, 1998).

This study found that it was common for participants to experience more than one emotion at once in the lead-up to suicide and/or violence, which could be a distressing and confusing experience. This finding is supported by literature which purports that ‘emotion coupling’, or multi-level emotional experiences, can serve to confuse and/or further distress the individual, due to not knowing how to respond to or understand the experience of two simultaneous emotions (Aaker et al., 2008; Fox et al., 2013; Larsen & McGraw, 2014). The current study presents a novel finding in the application of theories of emotion coupling to experiences of suicide and violence.

Of importance, this study found a large degree of overlap in the emotions experienced before acts of *both* suicide and violence, suggesting the existence of a common pathway to suicide and violence. Research into the notion of ‘dual harm’ (harm to self and others) is expanding. Recently, the cognitive-emotional model of dual harm was proposed (Shafti et al., 2021), and central to this framework is the role of emotions, therefore lending support to the present findings.

Finally, this study has also shed light on the complex interplay between emotions in the lead-up to suicide and/or violence. Participants stated that fear was often at the root of other emotions experienced. This finding is supported by several theories of emotion which state that primary emotions such as fear are evolutionary universal (Lewis, 2000; Rosenberg & Ekman, 2020; Sroufe, 1979). In contrast, secondary emotions are created due to our social interactions with others (Kemper, 1978, 1991), thus lending support to the present findings. Furthermore, this study found that participants spoke about a common ‘cycle’ of emotions in relation to both suicide and violence. This finding is supported by theories of ‘emotional chaining’ whereby the individual’s appraisal of the current emotion forms a link into the next emotion, which is triggered by the appraisal (Ellis & Grieger, 1986).

### 4.2. Strengths, limitations and areas for future research

This study has utilised a novel methodology to overcome pre-existing barriers in relation to data collection with male prisoners with suicidal or violent histories. Using participatory visual methodologies in this study led to a greater rapport between researcher and participant. This could be because of the ability of participatory visual methodologies to redress the power imbalance inherent between researcher and participant, particularly within a forensic context (Bates et al., 2017; Jurkowski & Paul-Ward, 2007). Moreover, visual methodologies are known to lead to better quality and more trustworthy data due to; uncovering new understandings of phenomena led by participants, not researchers (Bates et al., 2017; Holloway & Galvin, 2016; Hurworth, 2004), increasing engagement with the interview process (Collier, 1957) and facilitating triangulation of data (Patton, 2002). Not only has this led to new discoveries in terms of the specific topic area, but more widely this study has pioneered a more creative data collection method with a unique population. The use of this novel methodology not only strengthens the results of this study, but also has implications for future studies in a range of fields. Participatory visual methodologies should be considered in studies where verbal articulation of emotions is difficult. This can include samples such as in this study (male prisoners who score highly in traits of alexithymia), but could also extend to other populations such as children, or individuals with learning disabilities or neurodevelopmental conditions such as autism spectrum disorder.

A further strength of this study is the involvement of those with lived experience throughout the study. Indeed, it was the members of a lived experience advisory group who first raised the challenges of collecting interview data from individuals who scored highly on a measure of difficulty identifying and communicating emotions. This group then helped to develop the participatory drawing task and piloted the topic guide themselves to give feedback. Such inclusion in this study was of utmost importance to ensure that the outcomes are relevant and relatable to male prisoners.

Despite these strengths, a limitation of the present study is its narrow focus. Interviews were conducted with only nine participants, all in the North West of England, and in only two different categories of prisons. Whilst qualitative research does not endeavour to provide ‘representative’ data of entire populations, it is prudent to interpret the findings of this study within the narrow context with which it occurred.

Related to this, future research could look to expand on the exploratory findings presented here, using complimentary quantitative methods. For instance, using methods such as the Card Sort Task for Self-harm (Townsend et al., 2016), it may be possible to create a more accurate chronology of the emotional experiences felt in the months, weeks, days and hours leading to suicide/violence. Similarly, experience sampling methodologies may help to uncover these questions (Pratt & Taylor, 2019). This focus on emotional experiences is currently not explored in the literature and this would therefore be a valuable addition to the current knowledge base.

Furthermore, future research may wish to elucidate the relationship between specific emotions, or sets of emotions, with thoughts and behaviours in relation to suicide and violence. As aforementioned, though several models of suicide and violence include emotional factors, these are primarily discussed in relation to the development of suicidal or violent *thoughts* as opposed to acts. The findings here, however, suggest a complex interplay between emotions and the decision and motivation to act on suicidal or violent thoughts. Future research may also wish to focus on more explanatory and evaluative research questions, for instance exploring the efficacy of specific interventions on the emotions outlined as being associated with suicide and/or violence.

### 4.3. Clinical implications

There are a number of implications from these findings that ultimately could reduce suicide and violence within male prisons. First, provisions should be made which encourage male prisoners to discuss their emotions with others. This will likely entail a cultural shift within the prison environment and will require challenges to the traditional notion of masculinity. Second, psychoeducation could be given to male prisoners at risk of suicide and/or violence which aims to inform how to recognise and communicate emotions. Such a program of psychoeducation should specifically focus on teaching prisoners to identify when they are feeling ‘inner turmoil’ or ‘an absence of emotions’ and encourage them to notify staff members of this. This could be an important indicator that a prisoner is at risk of acting on thoughts of suicide and/or violence.

The findings of this study also highlight the value of offering non-verbal approaches to treatment for those who struggle with identifying and discussing their emotions. Previous trials have determined art therapy to be successful within a prison population (Gussak, 2009) and it has also been found that non-verbal therapies such as mindfulness and exercise-based theory are successful in the prison population (Auty et al., 2017; O’toole et al., 2018). The findings of this study lend further encouragement to the use of non-verbal interventions in a prison environment.

**Role of the funding source**: This study was conducted as part of the first author’s PhD funded by the Medical Research Council Doctoral Training Program (MRC-DTP) and the University of Manchester President’s Doctoral Scholar Award. The funders had no role in the study design, collection, analysis or interpretation of the data, writing the manuscript, or the decision to submit the study for publication.
